# Structural and electronic properties of the active site of [ZnFe] SulE

**DOI:** 10.3389/fmolb.2022.945415

**Published:** 2022-10-10

**Authors:** Samah Moubarak, Yvonne Rippers, Nadia Elghobashi-Meinhardt, Maria Andrea Mroginski

**Affiliations:** ^1^ Technische Universität Berlin, Institut für Chemie, Berlin, Germany; ^2^ Freie Universität Berlin, Fachbereich Physik, Berlin, Germany

**Keywords:** density functional theory calculations, structural biology, computational modeling, metalloenzyme active site, electronic properties, sulerythrin, quantum mechanical/molecular mechanical (QM/MM) computations

## Abstract

The function of the recently isolated sulerythrin (SulE) has been investigated using a combination of structural and electronic analyses based on quantum mechanical calculations. In the SulE structure of Fushinobu et al. (2003), isolated from a strictly aerobic archaeon, *Sulfolobus tokadaii*, a dioxygen-containing species was tentatively included at the active site during crystallographic refinement although the substrate specificity of SulE remains unclear. Studies have suggested that a structurally related enzyme, rubrerythrin, functions as a hydrogen peroxide reductase. Since SulE is a truncated version of rubrerythrin, the enzymes are hypothesized to function similarly. Hence, using available X-ray crystallography data (1.7 Å), we constructed various models of SulE containing a ZnII–Fe active site, differing in the nature of the substrate specificity (O_2_, H_2_O_2_), the oxidation level and the spin state of the iron ion, and the protonation states of the coordinating glutamate residues. Also, the substrate H_2_O_2_ is modeled in two possible configurations, differing in the orientation of the hydrogen atoms. Overall, the optimized geometries with an O_2_ substrate do not show good agreement with the experimentally resolved geometry. In contrast, excellent agreement between crystal structure arrangement and optimized geometries is achieved considering a H_2_O_2_ substrate and FeII in both spin states, when Glu92 is protonated. These results suggest that the dioxo species detected at the [ZnFe] active site of sulerythrin is H_2_O_2_, rather than an O_2_ molecule in agreement with experimental data indicating that only the diferrous oxidation state of the dimetal site in rubrerythrin reacts rapidly with H_2_O_2_. Based on our computations, we proposed a possible reaction pathway for substrate binding at the ZnFeII site of SulE with a H_2_O_2_ substrate. In this reaction pathway, Fe or another electron donor, such as NAD(P)H, catalyzes the reduction of H_2_O_2_ to water at the zinc–iron site.

## Introduction

**FIGURE 1:**
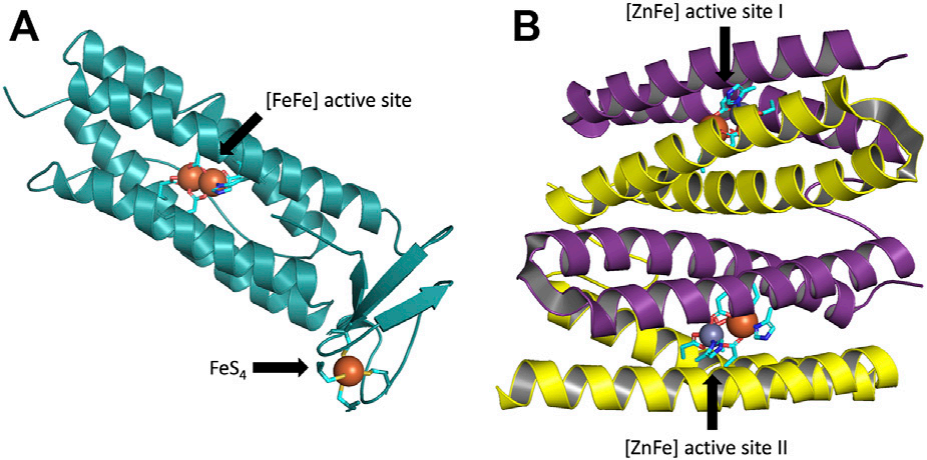
**(A)** One subunit of the rubrerythrin tetramer with its four-helix bundles containing a N-terminal diiron active site and a C-terminal rubredoxin-like FeS_4_ motif. The structure taken from PDB: 1RYT, resolution 2.1 Å (DeMaré et al., 1996). **(B)** Homodimeric protein SulE, containing the N-terminal domain of rubrerythrin, with its four-helix bundles and active sites I and II, each containing a [ZnFe] center. The structure taken from PDB: 1J30, resolution 1.7 Å (Fushinobu et al., 2003). Subunit A is colored in yellow and subunit B in purple. Carbon atoms coordinating the active center are colored in cyan, iron atoms in green, zinc atoms in gray, oxygen atoms in red, nitrogen atoms in blue, and sulfur atoms in yellow.

The present work addresses the structure of the active site of [ZnFe] sulerythrin (SulE) isolated from a strictly aerobic archaeon, *Sulfolobus tokadaii*, which is the smallest and first aerobic protein member of the rubrerythrin family (Wakagi, 2003). Rubrerythrin, which was first found in *Desulfolvibrio vulgaris,* is a non-heme iron homodimeric protein involved in oxidative stress tolerance in anaerobic bacteria and archaea (Sztukowska et al., 2002). Rubrerythrin contains both a hemerythrin-like binuclear iron cluster and a rubredoxin-like FeS_4_ center in each subunit (LeGall et al., 1988; Prickril et al., 1991) (Figure 1A). Hemerythrin-like proteins are generally characterized by their ability to reversibly bind oxygen through their binuclear non-heme iron centers (Alvarez-Carreno et al., 2018). Rubredoxin-like proteins are characterized by their [Fe(Cys)_4_] cluster (Kiss et al., 2019) (Figure 1A). SulE contains only the N-terminal domain of rubrerythrin, hence, the C-terminal domain containing a FeS_4_ motif of rubrerythrin is lacking (Wakagi, 2003) (Figure 1B).

So far, the physiological function of SulE remains unknown. SulE may play a role in oxygen binding or defense against oxidative stress (Wakagi, 2003). Consequently, SulE could be interesting for biocatalysts and smart materials such as semiconductors to enable electrical connection or to optimize stability and reaction conditions (Deutz et al., 2001; Varadan et al., 2006).

**FIGURE 2:**
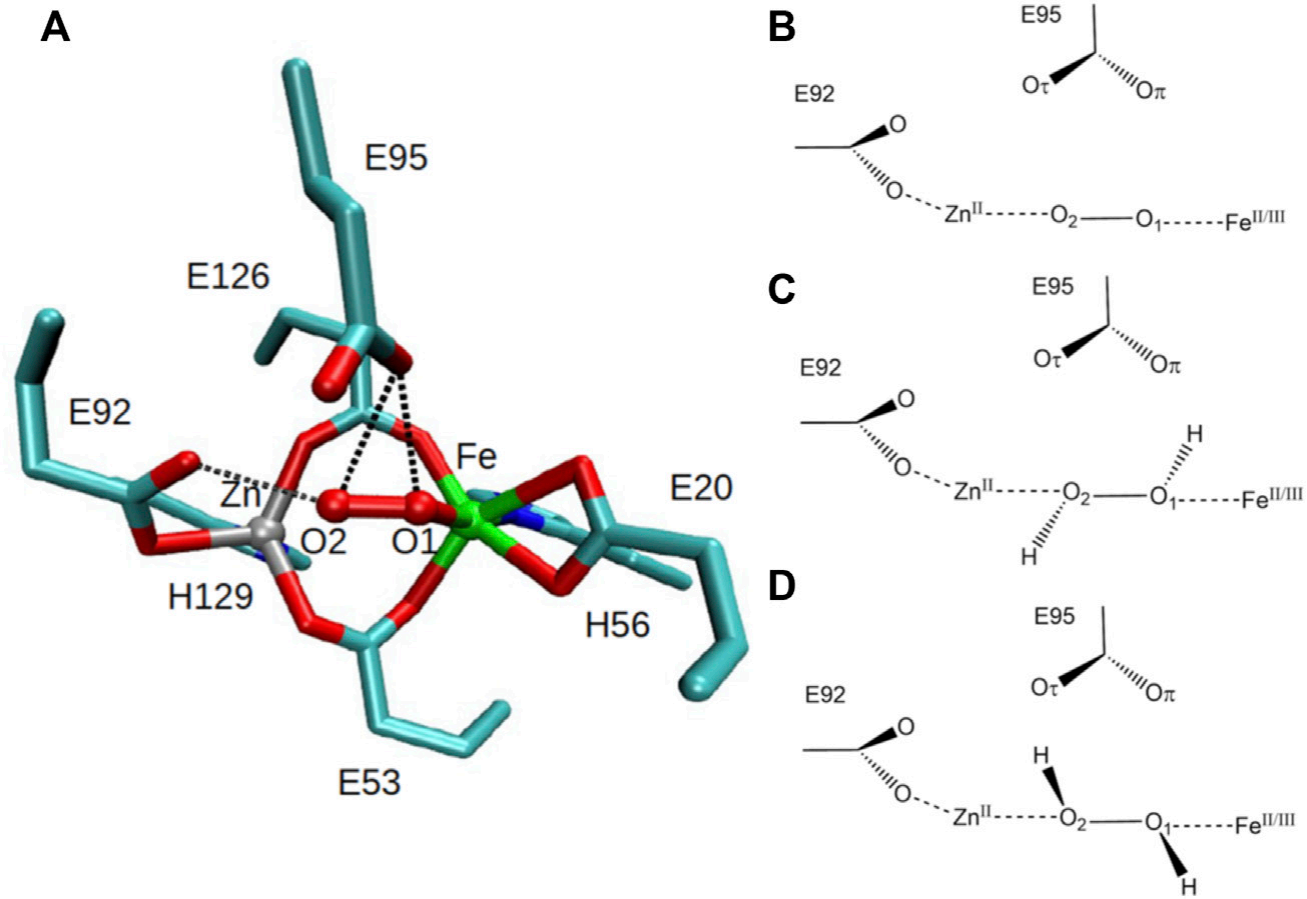
Left: **(A)** octahedrally coordinated iron and tetrahedrally coordinated zinc bimetallic active site of SulE with bridging bidentate carboxylates from Glu53 and Glu126, terminal bidentate carboxylates from Glu20 and Glu92, and with terminal histidine ligands, His56 and His129 [PDB: 1J30, resolution 1.7 Å (Fushinobu et al., 2003)]. Residues Glu95 and Glu92 are close to the putative dioxygen-containing species, resulting in short hydrogen-bonding distances (dotted lines) between Glu95 and O1/O2 and Glu92 and O2. Carbon atoms are colored in cyan, iron atom in green, zinc atom in gray, oxygen atoms in red, and nitrogen atoms in blue. Right: schematic view of the structural models of the active site of [ZnFe] SulE with O_2_ as a substrate **(B)**, H_2_O_2_ as a substrate in configuration “a” **(C)** and in configuration “b” **(D)**. Iron is modeled in oxidation states II and III.

The structure of SulE reveals a dimer with two subunits, each containing a four-helix bundle with a bimetallic active site which differs in the variation of the metal centers M1 and M2 (Wakagi, 2003) (Figure 1B). Here, as proposed by Fushinobu et al. (2003), the bimetallic center consists of a Zn atom and an Fe atom. Brown crystals identified in the protein sample, in which no change of color was observed after data collection, led to the crystallographic refinement of the Fe ion in a +III oxidized state (Fushinobu et al., 2003). The Fe atom is octahedrally hexacoordinated by the terminal bidentate carboxylates from Glu20, one of the bridging bidentate carboxylates from Glu53 and Glu126, respectively, imino nitrogen (Nδ1) from His56, and by a terminal oxygen atom of a putative dioxygen species (Fushinobu et al., 2003) (Figure 2). The Zn ion in the bimetallic active site of SulE is tetrahedrally coordinated by a terminal monodentate carboxylate from Glu92, imino nitrogen (Nδ1) from His129, and by one of the bridging bidentate carboxylates from each Glu53 and Glu126, respectively (Fushinobu et al., 2003). Thus, Glu53 and Glu126 form bridges between the metallic centers (Fushinobu et al., 2003).

The active site of SulEs with its various coordinated ligands in the binuclear metal center is asymmetric independent of the nature of the metal ions (Fushinobu et al., 2003; Jeoung et al., 2021; Lennartz et al., 2022). In particular, in [ZnFe] SulE, the occupancies of Zn atoms (0.87 and 0.80 for sites I and II, respectively) (Figure 1B) in the crystal structure of SulE are lower than those of the Fe atoms (1.0), suggesting that iron is more abundant in the bimetallic site than zinc (Fushinobu et al., 2003). In the study of Fushinobu et al. (2003), the dioxygen-containing species was tentatively included at the active site during crystallographic refinement due to the aerobic preparation of the SulE crystal throughout the purification and crystallization steps. Since the refined occupancies of the putative dioxygen-containing species do not greatly differ from those of Zn atoms, only the ZnII-containing molecules in the crystal might be able to bind the ligand if those molecules are composed of oxygen atoms or atoms with similar scattering capabilities (Fushinobu et al., 2003). Another possibility instead of the putative dioxygen species could be that two water molecules or ions with similar partial occupancies are bound (Fushinobu et al., 2003). A third alternative could be that partial reduction in the X-ray beam formed a mixture of FeII and FeIII, resulting in a FeII–O_2_ adduct in the crystal (Fushinobu et al., 2003). However, crystallographic resolution (1.7 Å) is not high enough to distinguish between these possibilities (Fushinobu et al., 2003). The substrate specificity of [ZnFe] SulE, therefore, remains unclear.

Two residues, namely, Glu95 and Glu92, are in hydrogen bonding distance to the putative dioxygen-containing species. The short hydrogen-bonding distances between Glu95 and O1/O2 [2.6 Å to both O1 and O2 in sites I and II, respectively (Fushinobu et al., 2003)] and between Glu92 and O2 [2.5 Å and 2.9 Å in sites I and II, respectively (Fushinobu et al., 2003)] of the putative dioxygen-containing species (Figure 2) indicate that either the Glu residue or the ligand atom is protonated, both of which are unlikely at pH 7.5 (Fushinobu et al., 2003). Alternatively, the short hydrogen bonds may arise due to disordered water as already suggested (Fushinobu et al., 2003).

As mentioned earlier, one assumption concerning the nature of the dioxygen-containing species in [ZnFe] SulE is that it could be an O_2_ molecule. However, Kurtz (2006) showed that rubrerythrin preferentially binds hydrogen peroxide over dioxygen at the iron-containing active sites (Coulter and Kurtz, 2001). Consequently, rubrerythrin functions as a hydrogen peroxide reductase by scavenging hydrogen peroxide and reducing it to water (Kurtz, 2006). Since strict anaerobes cannot use dioxygen as a terminal respiratory electron acceptor, the *D. vulgaris* species uses sulfate instead (Kurtz, 2006). However; SulE, being an aerobic protein, does not contain sulfate, but can use dioxygen as a terminal respiratory electron acceptor. As an electron donor, one of the reduced pyridine nucleotide phosphates, NAD(P)H, is often used in aerobic bacteria (Kurtz, 2006). In such cases, H_2_O_2_ is scavenged by NAD(P)H, catalyzed by a peroxidase (such as rubrerythrin), and reduced with two electrons to water (Kurtz, 2006).

$$\text{NAD}\left(P\right){H+H}^{+}{+\;2e}^{-}{\;+\;H}_{2}O_{2}\to \text{NAD}{\left(P\right)}^{+}{\;+\;2\;H}_{2}O.$$ (1)

Indeed, peroxidase (hydrogen peroxide reductase) activity has been reproducibly observed in rubrerythrin (Coulter et al., 2000; Coulter and Kurtz, 2001; Weinberg et al., 2004). However, only the diferrous oxidation level of the diiron site reacts rapidly with hydrogen peroxide (Weinberg et al., 2004), whereas the diferric site shows little or no such reactivity (Pierik et al., 1993; Coulter et al., 2000). On the other hand, the active site of rubrerythrin might divert the reaction between the ferrous ion and hydrogen peroxide, in a so-called Fenton reaction (Kurtz, 2006). Fenton chemistry describes the oxidization of an aqueous ferrous ion by hydrogen peroxide to a ferric ion, thus generating a highly oxidizing hydroxyl radical (Eq. 2) (Pignatello et al., 1999; Kurtz, 2006).

$${\text{Fe}}^{2+}\left(\text{aq}\right){\;+\;H}_{2}O_{2}\to {\text{Fe}}^{3+}\left(\text{aq}\right)\;{+\;\text{OH}}^{-}+\cdot \text{OH}.$$ (2)

The ferric ion can be then re-reduced to iron(II) by another H_2_O_2_ molecule, resulting in the formation of a hydroperoxyl radical and a proton (Eq. 3) (Pignatello et al., 1999; Kurtz, 2006).

$${\text{Fe}}^{3+}\left(\text{aq}\right){\;+\;H}_{2}O_{2}\to {\text{Fe}}^{2+}\left(\text{aq}\right){\;+\;\text{HO}}_{2}{\cdot +H}^{+}.$$ (3)

In a disproportionation reaction of hydrogen peroxide (Eqs 2,3), two different oxygen-radical species arise and a water molecule is formed (Eq. 4) (Pignatello et al., 1999; Kurtz, 2006).

$${2H}_{2}O_{2}\to H_{2}{O+\text{HO}}_{2}\cdot +\cdot \text{OH}.$$ (4)

Since SulE is a truncated version of rubrerythrin, the enzymes are hypothesized to function similarly. Consequently, SulE may bind hydrogen peroxide.

Until now, the structural and mechanistic details of SulE have not been investigated computationally, although a range of spectroscopic experiments have been performed. The molecular mass determined by sodium dodecyl sulfate-polyacrylamide gel electrophoresis (SDS-PAGE) (15.8 kDa), time-of-flight mass spectrometry (TOF-MS) (16.3 kDa) and gel filtration chromatography (GFC) (34.5 kDa), suggests that the protein is a homodimer (Wakagi, 2003). Ultraviolet-circular dichroism (UV-DC) shows that the protein is mostly composed of α-helices (Wakagi, 2003). In the structure of Fushinobu et al. (2003), the metal content was determined by inductively coupled plasma atomic emission spectrometry (ICP-AES), indicating that one of the two Fe atoms in the diiron center is replaced by Zn (Wakagi, 2003). Anomalous X-ray scattering (AXRS) indicates that the metal site of SulE is inverted compared to the positions of Fe and Zn in the native rubrerythrin, as isolated from *D. vulgaris* under aerobic conditions (Fe/Zn-Rbr_aero_) (Fushinobu et al., 2003). Absorption spectra indicate that SulE, as isolated, binds O_2_ (Wakagi, 2003). Furthermore, a recent crystallographic and biochemical study by Jeoung et al. (2021) reports the structure and hydrogen peroxide reactivity of four homobimetallic SulE variants: diMn(II)-, diFe(II)-, diCo(II)-, and diNi(II)- SulE (Jeoung et al., 2021). The authors show that all four metals bind sulerythrin with high affinity and that all four SulE variants react with H_2_O_2_ albeit with different turnover rates (Pierik et al., 1993). In a further study of Lennartz et al. (2022), SulE was reconstituted with ferrous iron (diFe–SulE) and treated with H_2_O_2_, thus, analyzed by a spatially resolved anomalous dispersion (SpReAD) refinement, a method to usually determine the redox states of metals in iron–sulfur cluster-containing proteins (Lennartz et al., 2022). The analysis indicates that each monomer in diFe–SulE coordinates two iron ions, one of which is in a more reduced state than the other (Lennartz et al., 2022).

To test Kurtz’s hypotheses regarding the binding preference of the active site in such enzymes, we investigate here both O_2_ and H_2_O_2_ as possible oxygen-containing substrates of SulE, as well as the electronic nature of the iron species present in the catalytic site. Thus, the present work reports on a series of quantum chemistry–based calculations describing the structural and electronic properties of models of the bimetallic active site of SulEs with two different dioxygen-containing species as substrates, namely, O_2_ and H_2_O_2_ (Fushinobu et al., 2003). As depicted in Figure 2, the structural models were constructed with a Zn(II) ion and either an Fe(II) or an Fe(III) ion at the active site, according to the structural data of Fushinobu et al. (2003). Although experimental and computational data indicate that nonheme iron-containing complexes are stable in the high-spin state (Cappillino et al., 2012a; Cappillino et al., 2012b), our preliminary computations indicated that a low Fe spin could also be relevant. Therefore, we investigated both high (S = 2 for FeII and S = 5/2 for FeIII) and low (S = 0 for FeII and S = 1/2 for FeIII) spin states for Fe. In addition, the protonation states of Glu92, Glu95, as well as two possible structural isomers of the H_2_O_2_ substrate and their preferential arrangement at the catalytic center have been considered. These quantum chemical calculations not only contribute to identify the nature of the dioxo ligand at the active site of [ZnFe] SulEs, but they also shed light onto their catalytic mechanism, thereby providing valuable information for rational design of new four-helix bundled enzymes with novel catalytical properties.

## Materials and methods

### Density functional theory calculations

Atomic coordinates of heavy atoms were extracted from PDB ID 1J30 from Fushinobu et al. (2003) (resolution 1.7 Å). Hydrogen atoms were added with GaussView6 (Dennington et al., 2016). The quantum chemical models include all atoms in the [ZnFe] catalytic center, the dioxo substrate, and amino acids in the first coordination sphere of the bimetal site, namely, the side chains of residues Glu53, Glu126, Glu20, Glu92, His56, His129, and Glu95 (Figure 2). In total, 28 models of the active site of [ZnFe] SulE were constructed. They differ in 1) the chemical nature of the dioxo species (O_2_, H_2_O_2_), 2) oxidation levels (+II, +III) and electronic spin states (high spin and low spin) of the iron ion, 3) protonation states of the Glu92 and Glu95, and 4) configuration of the H_2_O_2_ ligand (“a” and “b” configurations).

**FIGURE 3:**
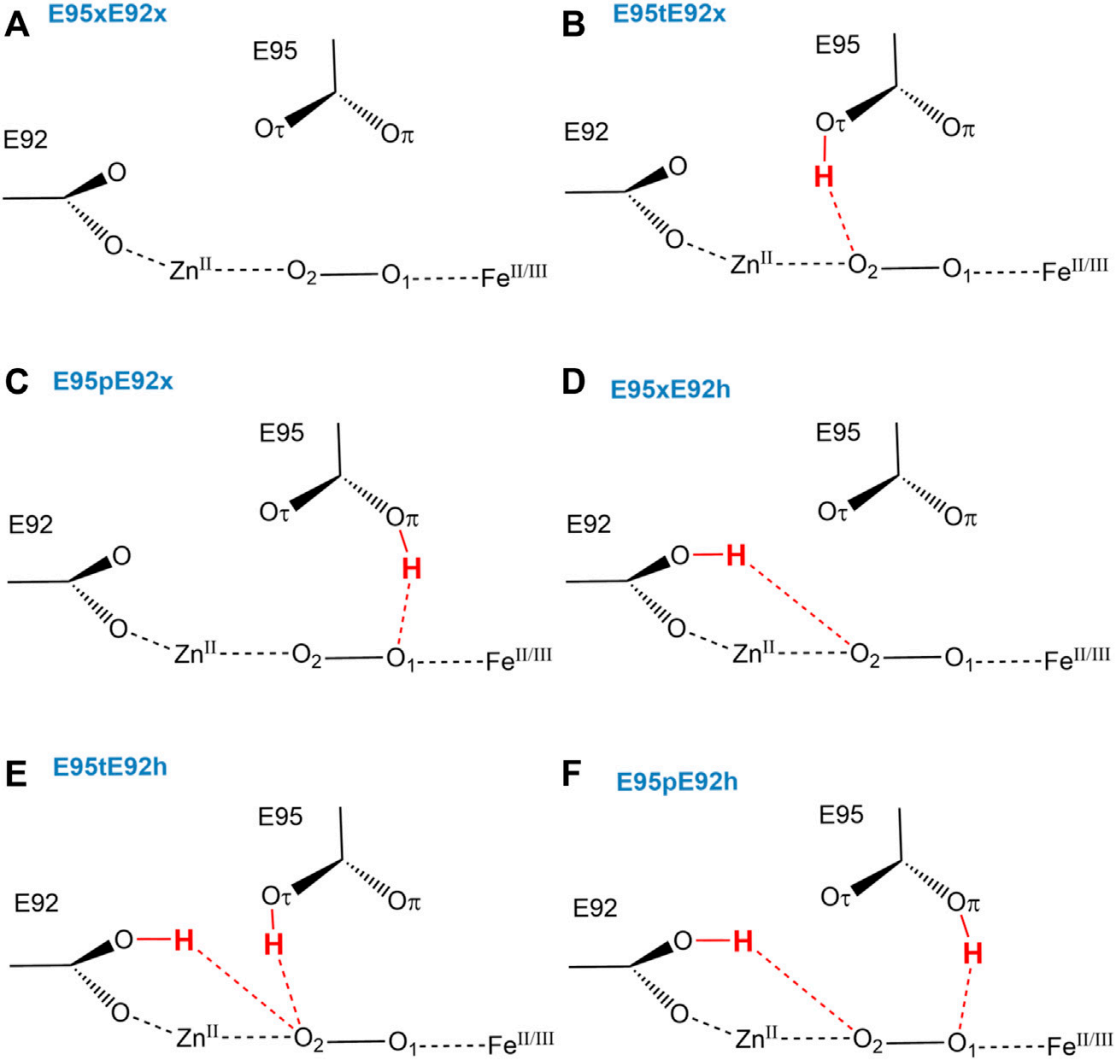
Schematic view of structural models **(A–F)** of the active site of [ZnFe] SulE with O_2_ as a substrate considering both Fe oxidation states (+II, +III) and different protonation states of Glu92 and Glu95.

**FIGURE 4:**
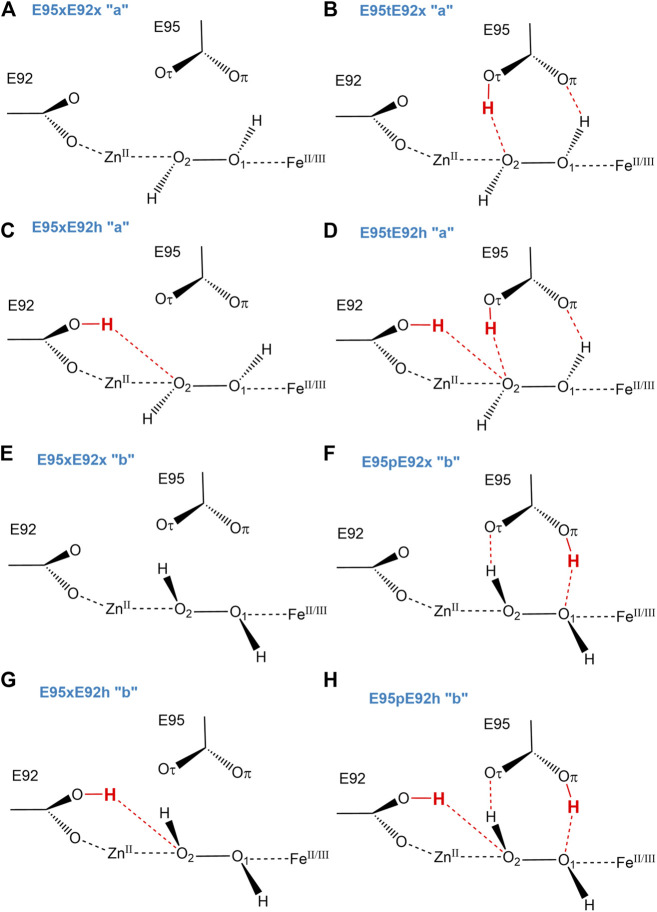
Schematic view of structural models of the active site of [ZnFe] SulE with H_2_O_2_ as a substrate considering both Fe oxidation states (+II, +III) are shown for the different protonation states of Glu92 and Glu95 and the two possible H_2_O_2_ configurations “a” **(A–D)** and “b” **(E–H)**.

Since Glu95 and Glu92 are in short hydrogen bonding distance to the putative dioxygen-containing species and Glu92 contains a monodentate carboxylate group instead of a bidentate carboxylate group, as it is the case for Glu20, Glu53, and Glu126 (Fushinobu et al., 2003); only Glu95 and Glu92 will be selected for a detailed consideration of their protonation states. The protonation states of Glu92 and Glu95 are described using the following nomenclature: first, the Oτ oxygen of the Glu92 carboxyl group can be either protonated (E92h) or deprotonated (E92x) in each configuration, since the Oπ oxygen is directly coordinated to the zinc metal site. Second, since the carboxyl group of Glu95 is located above the oxygen-containing substrate and orthogonal to the metal connection axis, there are three realistic protonation states for Glu95 in each configuration possible. Either the carboxyl group of Glu95 is completely deprotonated (E95x), or one of the two oxygens in Glu95 is protonated. Hence, the oxygen atom that is farther away from the oxygen-containing substrate can be protonated (E95t, t from “tau-” or “tele-,” IUPAC), or the oxygen atom which is closer to the oxygen-containing substrate can be protonated (E95p, p from “pi-” or “pros-,” IUPAC). The resulting models for a [ZnFe] active site of SulE with O_2_ as a substrate are shown in Figure 3, while those with H_2_O_2_ as a substrate are depicted in Figure 4. The zinc–iron complexes were calculated in both high- and low-spin states and for both Fe oxidation states, namely, FeII and FeIII. The electronic states of the 28 models investigated here are summarized in Table 1. The ZnII(d^10^) atom provides a total spin of S = 0 corresponding to a spin multiplicity of 2S + 1 = 1 to the considered region. The FeII atom in a high-spin state, therefore, leads to a total spin of S = 2 and a spin multiplicity of 2S + 1 = 5, whereas, for FeII in a low-spin state, a total spin of S = 0 and a spin multiplicity of 2S + 1 = 1 are obtained. Furthermore, the FeIII atom in a high-spin state leads to a total spin of S = 5/2 and a spin multiplicity of 2S + 1 = 6, whereas FeIII in a low-spin state gives a total spin of S = 1/2 and a spin multiplicity of 2S + 1 = 2. All models were treated with non-relativistic density functional theory (DFT) and the B3LYP (Becke, 1993) functional as implemented in Gaussian16 (Frisch et al., 2016). In this regard, the benchmark of DFT functional on zinc and iron complexes showed that B3LYP can predict spin states very well and provides optimized geometries which are comparable to those computed with M06L MN15 (Amin and Truhlar, 2008; Verma et al., 2017). A mixed basis set of def2-TZVP (Fe and Zn atoms) and 6-31G* (C, N, O, and H atoms) was used; this combination of basis sets has been tested and shown to be appropriate for geometry optimization of such metallo-enzymes (Siebert et al., 2015). All C_α_ atoms were saturated with dummy hydrogen atoms and their coordinates were kept fixed during geometry optimizations. Convergence criteria of 10^−5^ hartree/bohr RMSD have been applied for QM geometry optimizations. The calculations were carried out with Gaussian16 (Frisch et al., 2016). The thermodynamical stability of the resulting models was evaluated by comparing Gibbs energies G computed as a sum of electronic and thermal free energy using the thermochemistry tools implemented in Gaussian16 (Frisch et al., 2016). In addition, interaction free energies between the substrate and active site ∆_I_G were estimated by subtracting the Gibbs energies of the isolated active site model G(AS) and substrate G(S) from that of the active site–substrate complex G(AS-S): ∆_I_G = G(AS-S)—G(AS)–G(S).

**TABLE 1:** Total charge, total spin S, and spin multiplicity 2S + 1 for the QM system for each model of [ZnFe] SulE with both O_2_ and H_2_O_2_ as a substrate and for both Fe oxidation states (+II, +III), as well as, both high- and low-spin configurations.

Model	Total charge		Total spin S				Multiplicity			
	FeII	FeIII	FeII		FeIII		FeII		FeIII	
			High	Low	High	Low	High	Low	High	Low
[ZnFe]-O_2_										
E95xE92x	−1	0	2	0	5/2	½	5	1	6	2
E95tE92x	0	1	2	0	5/2	½	5	1	6	2
E95pE92x	0	1	2	0	5/2	½	5	1	6	2
E95xE92h	0	1	2	0	5/2	½	5	1	6	2
E95tE92h	1	2	2	0	5/2	½	5	1	6	2
E95pE92h	1	2	2	0	5/2	½	5	1	6	2
[ZnFe]-H_2_O_2_										
Conf. “a”										
E95xE92x	−1	0	2	0	5/2	½	5	1	6	2
E95tE92x	0	1	2	0	5/2	½	5	1	6	2
E95xE92h	0	1	2	0	5/2	½	5	1	6	2
E95tE92h	1	2	2	0	5/2	½	5	1	6	2
Conf. “b”										
E95xE92x	−1	0	2	0	5/2	½	5	1	6	2
E95pE92x	0	1	2	0	5/2	½	5	1	6	2
E95xE92h	0	1	2	0	5/2	½	5	1	6	2
E95pE92h	1	2	2	0	5/2	½	5	1	6	2

### Hybrid quantum mechanics/molecular mechanics calculations

**FIGURE 5:**
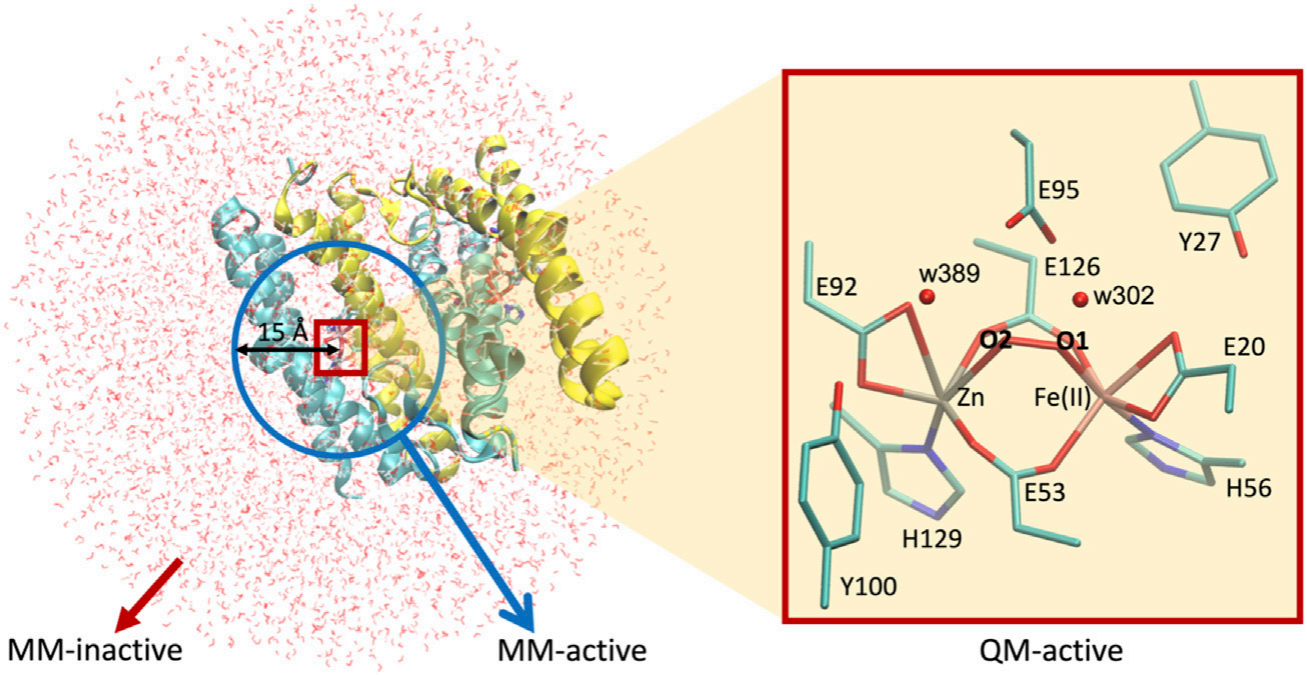
Setup for QM/MM calculations used in this work. Left, [ZnFe] SulE in a water droplet of 40 Å radius centered at the iron ion of chain A. Blue circle represents the 15 Å radius sphere containing atoms of the *MM-active* region while red box highlights the *QM-active* region. Atoms beyond the blue sphere belong to the *MM-inactive* region. Right, zoom in the QM-active region depicting all heavy atoms of the [ZnFe] SulE active site which are treated quantum mechanically.

This DFT-based computational study can be advanced by generating more complex and realistic models including explicitly the whole protein matrix surrounding the active site through hybrid quantum mechanics/molecular mechanics (QM/MM) methodology (Senn and Thiel, 2007). Thus, we applied this approach to further investigate the effect of the protein environment on the structural and electronic properties of the dimetallic site. Since these calculations are computationally demanding, the QM/MM approach was only applied to the nine most favorable models resulting from the analysis of the quantum chemical results, namely, (*vide infra*): E95xE92x (FeII—low spin) active site with O_2_, E95tE92x (FeII—low spin and high spin) active site with O_2_, E95xE92h (FeII—high spin) active site with O_2_, E95tE92x (FeII—low spin and high spin) active site with H_2_O_2_ in configuration “a,” E95xE92h (FeII—low spin and high spin) active site with H_2_O_2_ in configuration “a,” and E95tE92h (FeII—low spin) active site with H_2_O_2_ in configuration “a.” The initial Cartesian coordinates of all heavy atoms of the protein were extracted from the SulE crystal structure (PDB entry: 1J30) (Fushinobu et al., 2003). Hydrogen atoms were modeled using the *hbuild* tool of the CHARMM (V. 45b2) (Brooks et al., 2009) package with the CHARMM36 force field (Huang and MacKerell, 2013). His129 and His56 were protonated at position Nε due to possible interactions with the [ZnFe] center. Unless otherwise specified, protonation of amino acid side chains was according to the standard assignment for pH 7. The entire dimeric protein, including the [ZnFe] catalytic centers and crystal water molecules, were then solvated in a TIP3P water box with sodium chloride ions (0.1 M). Short energy minimizations steps using periodic boundary conditions were performed to optimize the hydrogen bond network at the protein–solvent interface (Brooks et al., 2009). All atoms within a 40 Å sphere centered on the iron ion of the [ZnFe] center of chain A (Figure 5) define the molecular system for the subsequent QM/MM calculations. Geometry optimization of the [ZnFe] active site of the nine SulE models and their immediate environment was performed using the QM/MM approach implemented in the modular program package ChemShell (Sherwood et al., 2003). This was performed by dividing the enzyme into three regions identified as QM, MM-active, and MM-inactive (Figure 5). Only the positions of atoms within the QM- and MM-active regions were optimized, whereas atoms in the MM-inactive region were held fixed. The QM region involves all atoms in the [ZnFe] catalytic center, as well as those belonging to amino acid side chains Glu95, Glu92, Glu20, Glu126, Glu53, His129, His56, Tyr27, and Tyr100. Two water molecules (w302 and w389) from the crystal structure of diMn-SulE (PDB-ID 7093) were also included in the QM region due to short hydrogen bonding distances to Glu95 and Glu92 (Figure 5). The MM-active region includes all atoms belonging to the protein and crystal water (oxygen) within a 15 Å radius of the Fe atom. Although the QM region was treated at the B3LYP/def2-TZVP (Fe and Zn atoms)/6-31G* (C, N, O, and H atoms) level of theory as in the QM computations described earlier, the MM-inactive region was described by a CHARMM36 force field. The covalent cuts at the QM/MM boundary were saturated with a hydrogen-like atom and the coupling between the QM- and the MM-active regions was modeled on the basis of the electrostatic embedding model with a charge shifted scheme (Senn and Thiel, 2007). The geometry optimization of the QM/MM system was performed using hybrid delocalized internal coordinates (HDLC) in combination with a limited memory L-BFGS quasi-Newton optimization algorithm (Billeter et al., 2000). Protein geometries were analyzed and depicted using the software Visual Molecular Dynamics (Humphrey et al., 1996).

## Results and discussion

### Density functional theory geometry optimizations of isolated [ZnFe] SulE with an O_2_ substrate

Geometry optimizations of the isolated structural models of the active site of [ZnFe] SulE with an O_2_ substrate show poor agreement with the experimentally resolved geometry as reflected by root-mean-square deviations (RMSD) relative to the crystal structure (Table 2). Notably, the active site itself experiences only minor deviations with respect to the crystal structure. However, in most of the performed geometry optimizations, significant displacement or reorientation of the O_2_ substrate takes place. The RMSD values listed in Table 2 indicate that the lowest RMSD values are predicted for the E95xE92x-FeII in the low-spin state, the E95tE92x models in both high- and low FeII spin states, as well as for the E95xE92h model in a high FeII spin. In the case of the [ZnFeIII] SulE models, the predicted RMSD values are significantly above 0.6 Å (Table 2). Hence, we assume that structural data reported by Fushinobu et al. (2003) were collected under experimental conditions in which the iron ion is not in a ferric state.

**TABLE 2:** RMSD [Å] of heavy atom positions relative to the crystal structure arrangement [PDB ID 1J30 Fushinobu et al. (2003)] of all 23 structural models of the [ZnFe] active site and the O_2_ substrate. For clarity, RMSD values below 0.5 Å are highlighted in bold.

Model	RMSD_{active site}^a^				RMSD_{O_2_}			
	FeII		FeIII		FeII		FeIII	
	High	Low	High	Low	High	Low	High	Low
E95xE92x	0.551	**0.435**	0.958	0.839	1.302	0.581	0.984	0.999
E95tE92x	**0.438**	**0.463**	1.018	0.665	1.490	0.739	3.447	0.742
E95pE92x	0.886	0.728	1.141	0.972	1.232	0.565	1.834	2.347
E95xE92h	**0.472**	1.066	0.755	—	0.557	**0.425**	0.887	—
E95tE92h	0.716	0.671	1.018	0.971	3.094	0.790	2.638	0.971
E95pE92h	0.981	1.115	1.005	0.964	0.555	0.650	2.644	2.664

a RMSD values [Å] for the active site (protein) heavy atoms and without the substrate.

**FIGURE 6:**
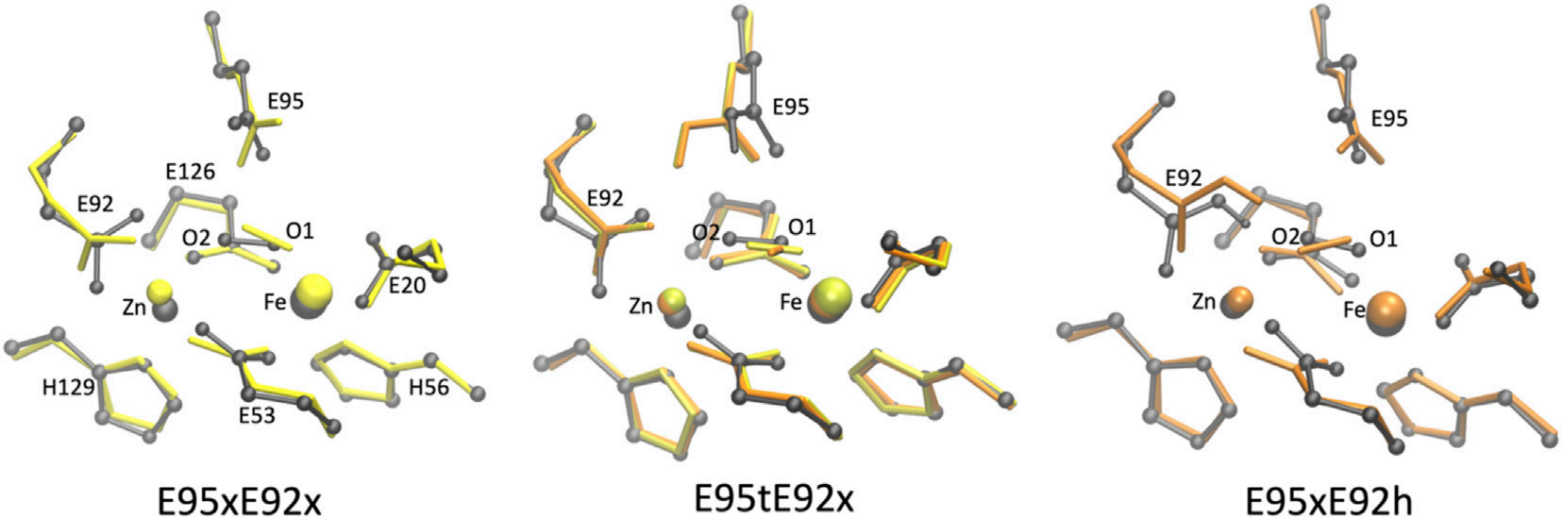
Superposition of the active site of the [ZnFe] SulE crystal structure (gray) and B3LYP/def2-TZVP/6-31G* optimized geometries of the four best structural models E95xE92x (left), E95tE92x (middle) and E95xE92h (right) with FeII center in a low-spin state (yellow) or high-spin state (orange) harboring an O_2_ substrate.

To investigate the structural properties of the [ZnFeII] catalytic site in greater detail, the optimized geometries of the structural models with the closest agreement with the crystal structure (RMSD < 0.5 Å) are superimposed in Figure 6.

As highlighted in Table 2, geometry optimizations of only four structural models of the [ZnFeII] site led to geometries in close agreement with the crystal structure (Figure 6). The Zn–Fe distance of 3.83 Å in the crystal structure is well reproduced by the E95xE92h high-spin model (3.71 Å) and is overestimated in about 0.4 Å in the other three structures (4.11 Å, 4.10 Å, and 4.03 Å for E95xE92x, E95tE92x low spin, and E95tE92x high spin, respectively). Although, the bond length of the O_2_ substrate predicted at around 1.27 ± 0.02 Å in all four models agrees well with the experimental crystallographic value of 1.25 Å, significant displacement or reorientation of it with respect to the two metal centers is observed upon geometry optimization as reflected by the large RMSD values predicted for the oxygen atoms (above 0.5 Å). Indeed, among these models, the distance to the iron center does not change drastically (Fe–O1_models_: 1.75–2.35 Å), while the distance to the Zn center does (Zn–O2_models_: 2.34–3.99 Å) compared to the crystallographic values (Fe–O1_crystal_: 2.12 Å and Zn–O2_crystal_: 2.74 Å). The best agreement is predicted for E95xE92h in the high-spin state with Fe–O1_model_: 2.35 Å and Zn–O2_model_: 2.34 Å (Supplementary Table S1).

Additional information on the conformational stability of the [ZnFe] active site can be gained by analyzing ground state differences between the Gibbs energy of high- and low Fe spin states of the same models. These values are listed in Table 3 for all [ZnFe] SulE active site models binding O_2_. Notably, all structural models with iron in a high-spin state converge to geometries that are energetically more favorable than the corresponding low-spin models, independent of the Fe oxidation state (Table 3).

**TABLE 3:** Gibbs energies, G, [Hartree] of all DFT optimized structural models of the [ZnFe] active site harboring an O_2_ substrate. ∆G represents the difference between Gibbs energies of high- and low-spin states for each structural model in kcal/mol units.

Model	Gibbs energy G [Hartree]		∆G [kcal/mol] (high spin–low spin)
	High spin	Low spin	
FeII			
E95xE92x	−5338.3384	−5338.2828	−35
E95tE92x	−5338.9062	−5338.8425	−40
E95pE92x	−5338.9170	−5338.8431	−46
E95xE92h	−5338.8486	−5338.8006	−30
E95tE92h	−5339.3162	−5339.2518	−40
E95pE92h	−5339.3177	−5339.2587	−37
FeIII			
E95xE92x	−5338.2481	−5338.2191	−18
E95tE92x	−5338.7048	−5338.6450	−38
E95pE92x	−5338.7033	−5338.6896	−9
E95xE92h	−5338.6519	—	—
E95tE92h	−5339.0197	−5339.0141	−4
E95pE92h	−5339.0198	−5339.0125	−5

In summary, the structural and energetical analysis of the DFT optimized models of the active site of the [ZnFe] SulE harboring an O_2_ molecule suggest that model E95xE92h with the high-spin state of the FeII center best resembles the crystallographic arrangement although the dioxygen moiety is slightly displaced with respect to their position in the crystal (RMSD for O_2_: 0.557). According to this model, the O_2_ substrate directly interacts with the Zn and Fe ions and it is additionally stabilized by H-bond interactions with the protonated Glu92 (distance O2-O_E92_: 2.65 Å). The carboxylic side chains of the other neighboring glutamates remain deprotonated.

### Density functional theory geometry optimizations of isolated [ZnFe] SulE with an H_2_O_2_ substrate

Following the suggestion of Kurtz (2006), we replaced O_2_ with H_2_O_2_ to probe the nature of the dioxygen-containing species (Coulter et al., 1999; Coulter et al., 2000; Weinberg et al., 2004). DFT geometry optimizations of the isolated active site of [ZnFe] SulE with an H_2_O_2_ substrate yield structures which show an overall better agreement with the crystallographic data than those predicted with O_2_ as the dioxygen-containing species, as reflected by the relatively low RMSD of heavy atom positions relative to the crystal geometry.

The RMSD values summarized in Table 4 illustrate that the structural deviations from the crystal structure are more significant for the “b” configuration of the hydrogen peroxide molecule than those for the “a” configuration. This is particularly true for models with an iron(+II) site, for which RMSD values of the active site and substrate alone lie mostly above 0.5 Å. In the case of models with “b” conformation of H_2_O_2_ and independent of the electronic state of the iron site, we observe a significant reorientation of the substrate with respect to the metal centers.

**TABLE 4:** RMSD [Å] of heavy atom positions relative to the crystal structure arrangement [PDB ID 1J30 Fushinobu et al. (2003)] of all 24 structural models of the [ZnFe] active site and the H_2_O_2_ substrate. For clarity, RMSD values below 0.5 Å are highlighted in bold.

Model	RMSD active site^a^ [Å]				RMSD H_2_O_2_ substrate [Å]			
	FeII		FeIII		FeII		FeIII	
	High	Low	High	Low	High	Low	High	Low
Conf. “a”								
E95xE92x	0.710	0.849	0.886	0.934	**0.397**	**0.333**	**0.404**	**0.430**
E95tE92x	**0.469**	**0.464**	0.892	0.907	**0.470**	**0.347**	**0.382**	**0.424**
E95xE92h	**0.307**	**0.352**	0.773	0.556	0.721	0.914	0.521	**0.470**
E95tE92h	0.688	**0.299**	1.002	0.930	2.014	0.744	0.873	0.834
Conf. “b”								
E95xE92x	1.303	0.908	**0.405**	**0.387**	0.985	1.010	0.841	0.605
E95pE92x	0.855	**0.428**	0.935	0.905	2.558	0.785	0.739	0.598
E95xE92h	1.151	0.851	**0.436**	0.758	0.847	0.879	1.086	1.813
E95pE92h	0.730	0.705	0.516	1.136	1.209	1.031	0.565	0.862

a RMSD values [Å] for the active site (protein) heavy atoms and without the substrate.

Table 4 also shows that the RMSD values decrease from models E95xE92x to E95xE92h in the case of the [ZnFeII] active site with an “a” configuration of H_2_O_2_. These values, which are slightly lower for a high-spin FeII than those for a low-spin FeII state, result from minor rotations of the bridging carboxylic side chains of Glu53, for the E95xE92h “a” model. Although the lowest RMSD of the heavy atom positions of the active site relative to the crystal arrangement of 0.299 Å is predicted for model E95tE92h “a” with protonated Glu95 and Glu92 residues, such configuration leads to a significant displacement of the position of the dioxo ligand (RMSD of 0.744 Å).

Unlike iron(+II) models, structural models with an iron(+III) active site show the lowest RMSD values when the H_2_O_2_ ligand lies in “b” configuration as predicted, particularly for models E95xE92x and E95xE92h (high spin) with RMSD values below 0.5 Å. These models, however, show large deviation of the position of the oxygen atoms of the substrate, with RMSD values above 0.6 Å. Therefore, these results suggest that in [ZnFe] SulE crystals (Fushinobu et al., 2003), the iron ion lies preferentially in an oxidation state of +II rather than +III, particularly in combination with the binding of an H_2_O_2_ substrate in configuration “a.”

**FIGURE 7:**
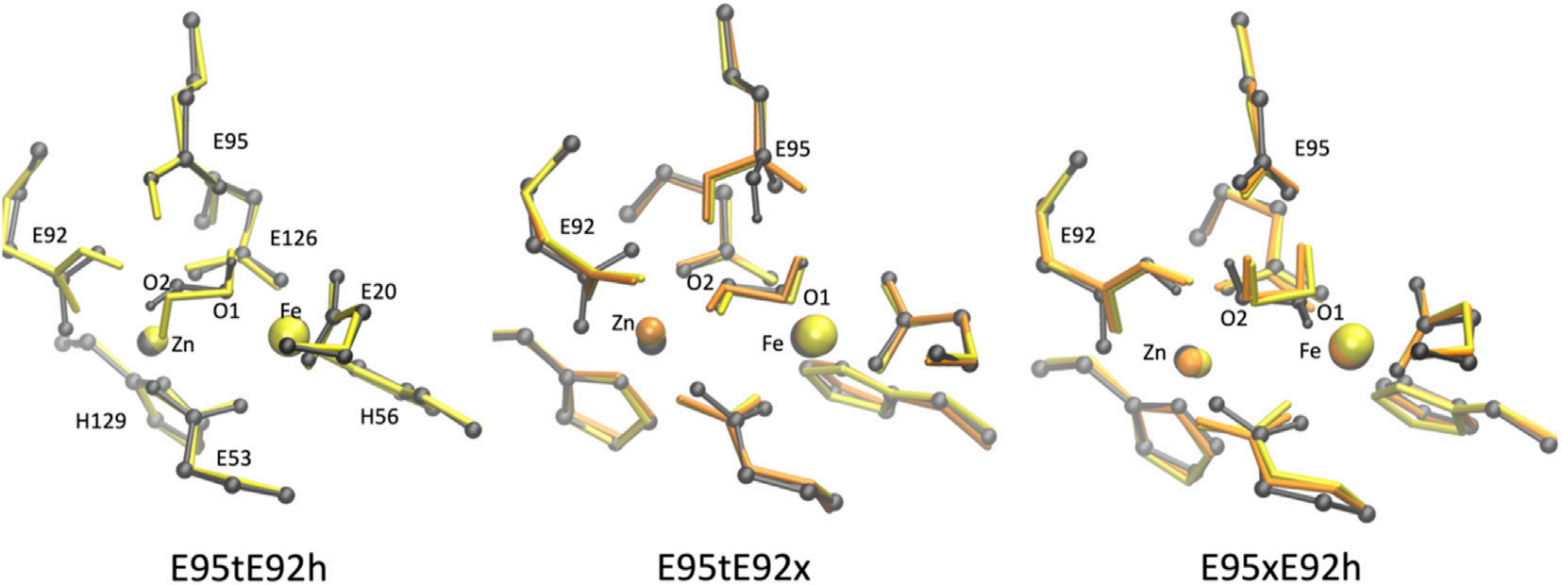
Superposition of the active site of the [ZnFe] SulE crystal structure (gray) and B3LYP/def2-TZVP/6-31G* optimized geometries of the five best structural models E95tE92h (left), E95tE92x (middle) and E95xE92h (right) with the FeII center in a low-spin state (yellow) or high-spin state (orange) harboring an H_2_O_2_ substrate in conformation “a.”

According to the predicted RMSD values, the E95tE92x models obtained in the “a” configuration show good agreement with the crystal structure for both high FeII spin (RMSD 0.469 Å) and low FeII spin (RMSD 0.464 Å) states (Table 4; Figure 7 middle). Nonetheless, in both electronic configurations, the side chain of Glu95 rotates slightly away from the starting geometry (displacement Oτ ∼1.30 Å), and hydrogen bonds are formed between the protonated Glu95 residue (E95t) and the O2 atom of the substrate (bond distance ∼1.84 Å), as well as between the Oπ atom from Glu95 and the proton of the substrate (bond distance ∼1.72 Å), presumably leading to an energetic stabilization of the substrate. Here again, the high-spin state is energetically lower than the low-spin state by 34 kcal/mol as predicted by the Gibbs energy calculations reported in Table 5.

**TABLE 5:** Gibbs energies, G, [Hartree] of all DFT optimized structural models of the [ZnFe] active site harboring an H_2_O_2_ substrate. ∆G represents the difference between Gibbs energies of high- and low- spin state for each structural model in kcal/mol units.

Model	Configuration “a”			Configuration “b”		
	G [Hartree]		∆G [kcal/mol]	G [Hartree]		∆G [kcal/mol]
	High spin	Low spin		High spin	Low spin	
FeII						
E95xE92x	−5339.6083	−5339.5553	−33	−5339.6290	−5339.5830	−29
E95t(p)E92x	−5340.1354	−5340.0819	−34	−5340.1475	−5340.0718	−48
E95xE92h	−5340.1201	−5340.0683	−33	−5340.1412	−5340.0720	−43
E95t(p)E92h	−5340.5571	−5340.4812	−48	−5340.5534	−5340.4943	−37
FeIII						
E95xE92x	−5339.5307	−5339.4946	−23	−5339.4621	−5339.4184	−27
E95t(p)E92x	−5339.9377	−5339.9035	−21	−5339.9228	−5339.8845	−24
E95xE92h	−5339.9389	−5339.9029	−23	−5339.8986	−5339.8594	−25
E95t(p)E92h	−5340.2447	−5340.2056	−25	−5340.2377	−5340.2122	−16

As mentioned earlier, E95xE92h “a” models also show good agreement with the crystal structure with RMSD of 0.307 Å for the high FeII spin and RMSD of 0.352 Å for the low FeII spin state (Figure 7, right). These two models are characterized by an anionic carboxylate side chain of Glu95 which forms H-bonds with the two hydrogens of H_2_O_2_. These interactions stabilize the substrate in a *cis*-like conformation. Although O2 of H_2_O_2_ interacts with the Zn center (Zn–O_2_ distance ∼3.34 Å in average for FeII in high- and low-spin states), O1 coordinates the Fe site (Fe–O1 distance ∼2.09 Å on average for FeII in high- and low-spin states) ( Supplementary Table S3). In addition, the protonated Glu92 carboxylic side chain is hydrogen bonded with the O_2_ atom of the substrate (distance ∼1.70 Å) thereby further stabilizing the substrate.

Among all active site models with iron in oxidation state II, the E95tE92h “a” model with low FeII spin shows the best structural agreement with crystallographic data (RMSD 0.299 Å) (Table 4; Figure 7, left). Here, the geometry of the catalytic site seems to be stabilized by hydrogen bonds between the protonated Glu92 side chain and O_2_ of the H_2_O_2_ substrate (distance 1.81 Å), between O_2_ of H_2_O_2_ and one oxygen atom of the Glu53 residue (distance 1.79 Å) and between O1 of H_2_O_2_ and Oπ of Glu95 (distance 1.66 Å). Interestingly, the same E95tE92h “a” model but with the high FeII spin state deviates significantly from the crystal structure (RMSD 0.688 Å) (Table 4). Not only a large displacement of H_2_O_2_ away from the starting geometry (distance Fe–O1∼3.74 Å, Supplementary Table S3) is predicted but also substantial reorientation of the carboxylic side chains of Glu95, Glu20, and His56 is observed. These results indicate that although simultaneous protonation of the carboxylic side chain of Glu95 and Glu92 is feasible, this state can only arise when the iron(+II) ion is found in a low-spin state, which is 48 kcal/mol higher in energy than its high-spin analog. Thus, despite the geometrical similarity with the crystal structure, a E95tE92h “a” structure with low FeII spin is energetically less favorable.

Taken together, the structural and energetical analysis of a set of models of the isolated [ZnFe] active site of sulerythrin harboring a hydrogen peroxide substrate indicates that a high-spin FeII E95xE92h model with a protonated Glu92 side chain best describes crystallographic data. The distance between the two metal ions and the O–O bond length of the H_2_O_2_ moiety are predicted at 3.79 Å and 1.47 Å, respectively, both in very good agreement with the experimental values of 3.83 Å and 1.79 Å (Fushinobu et al., 2003). Nonetheless, RMSD values for the H_2_O_2_ substrate is significant (RMSD >0.7 Å) for this model (Table 4), due to larger Zn–O_2_ distances (3.36 Å) compared to the crystallographic structure (exp. 2.74 Å).

### Interaction free energies between the substrate and [ZnFe] site

The structural analysis of the isolated model active sites was complemented by the computation of the interaction free energies between the dioxygen substrate and the dimetal active site (see *Materials and methods* section). Only the nine optimized structural models which showed the best agreement with the crystallographic structure were considered for these computations Tables 6, 7, suggesting that O_2_ binding to the bimetallic active site is strongly favored when Glu92 is protonated as in model E95xE92h with FeII in the high-spin state, as reflected by the negative interaction energy ∆_I_G of −4 kcal/mol. In models E95xE92x and E95tE92x, on the other hand, positive interaction energies have been predicted for O_2_. On the contrary, the binding of H_2_O_2_ with the bimetallic active site of SulE is favorable in all five structural models considered for the calculation independent of the redox state of the metal ions and the protonation state of the neighboring side chains, as reflected by the negative ∆_I_G values (Table 7). These substrate interaction energies for H_2_O_2_ are one order of magnitude higher than those predicted for O_2_ suggesting weaker binding of O_2_ to the bimetal center compared to H_2_O_2_. A similar observation has been reported for the *end-on* binding of O_2_ to the diiron center of trypanosome alternative oxidase based on QM/MM calculations (Yamasaki et al., 2021). Interestingly, protonation of Glu92 also favors substrate binding to the [ZnFeII] center as reflected by the high free energy values predicted for the **E95xE92h** model with FeII in high- as well as in low-spin states of −70 kcal/mol and −67 kcal/mol, respectively.

**TABLE 6:** Interaction free energies ∆_I_G [kcal/mol] for O_2_ in selected model structures of [ZnFeII] SulE.

Model	Spin state	Free energies [Hartree]			∆_I_G [kcal/mol]
		G_AS-S_	G_AS_	G_S_	
FeII					
E95xE92x	Low	−5338.2828	−5187.9692	−150.3328	12
E95tE92x	High	−5338.9062	−5188.5973	−150.3320	14
E95tE92x	Low	−5338.8425	−5188.5259	−150.3308	9
E95xE92h	High	−5338.8486	−5188.5148	−150.3273	−4

**TABLE 7:** Interaction free energies ∆_I_G [kcal/mol] for H_2_O_2_ in selected model structures of [ZnFeII] SulE. Only conformation “a” of H_2_O_2_ is considered.

Model	Spin state	Free energies [Hartree]			∆_I_G [kcal/mol]
		G_AS-S_	G_AS_	G_S_	
E95tE92x	High	−5340.1354	−5188.5688	−151.5241	−27
E95tE92x	Low	−5340.0819	−5188.5180	−151.5241	−25
E95xE92h	High	−5340.1201	−5188.4987	−151.5096	−70
E95xE92h	Low	−5340.0683	−5188.4517	−151.5096	−67
E95tE92h	Low	−5340.4812	−5188.9134	−151.5244	−27

### Effect of the protein environment:Quantum mechanical/molecular mechanical geometry optimizations

In order to assess the effect of the protein environment on the structural and electronic properties of the [ZnFe] SulEs, we performed hybrid QM/MM computations. The oxidation and spin states of iron as well as protonation of the glutamate side chains were chosen based on nine DFT models that reproduce well the structural features observed in the crystal structure: E95xE92x_O_2_ (low spin), E95tE92x_O_2_ (low spin and high spin), and E95xE92h_O_2_ (high spin); E95tE92h_H_2_O_2_ (low spin), E95tE92x_H_2_O_2_ (low spin and high spin), and E95xE92h_H_2_O_2_ (low spin and high spin). The starting geometry of the protein and metal ions for the QM/MM calculations was extracted from the crystal structure of [ZnFe] SulE (Fushinobu et al., 2003); the initial coordinates for the water molecules were taken from the crystal structure of diMn SulE (PDB entry 7O93) (Jeoung et al., 2021).

**FIGURE 8:**
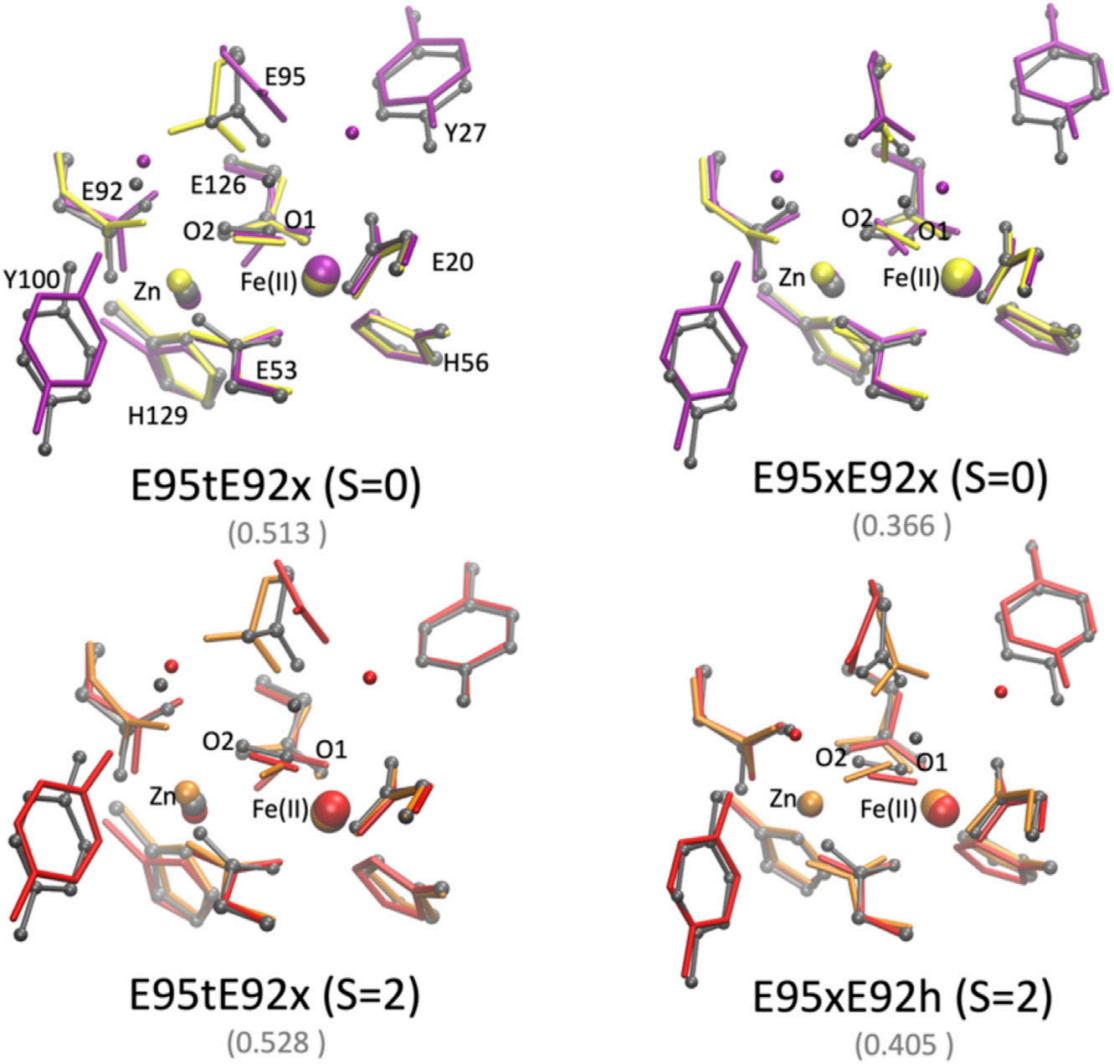
Superposition of the active site of the [ZnFe] SulE crystal structure (gray), B3LYP/def2-TZVP/6-31G* optimized geometries [Fe(II) low spin: yellow; Fe(II) high spin: orange] and QM/MM- optimized geometries [Fe(II) low spin: purple; Fe(II) high spin: red] of the four best structural models E95tE92x_low spin (top, left), E95xE92x_low spin (top, right), E95tE92x_high spin (bottom, left), and E95xE92h_high spin (bottom, right) with the FeII center harboring an O_2_ substrate. For clarity, hydrogen atoms are not shown. Corresponding RMSD values listed in Table 8 for the QM/MM models are given in gray fonts.

**FIGURE 9:**
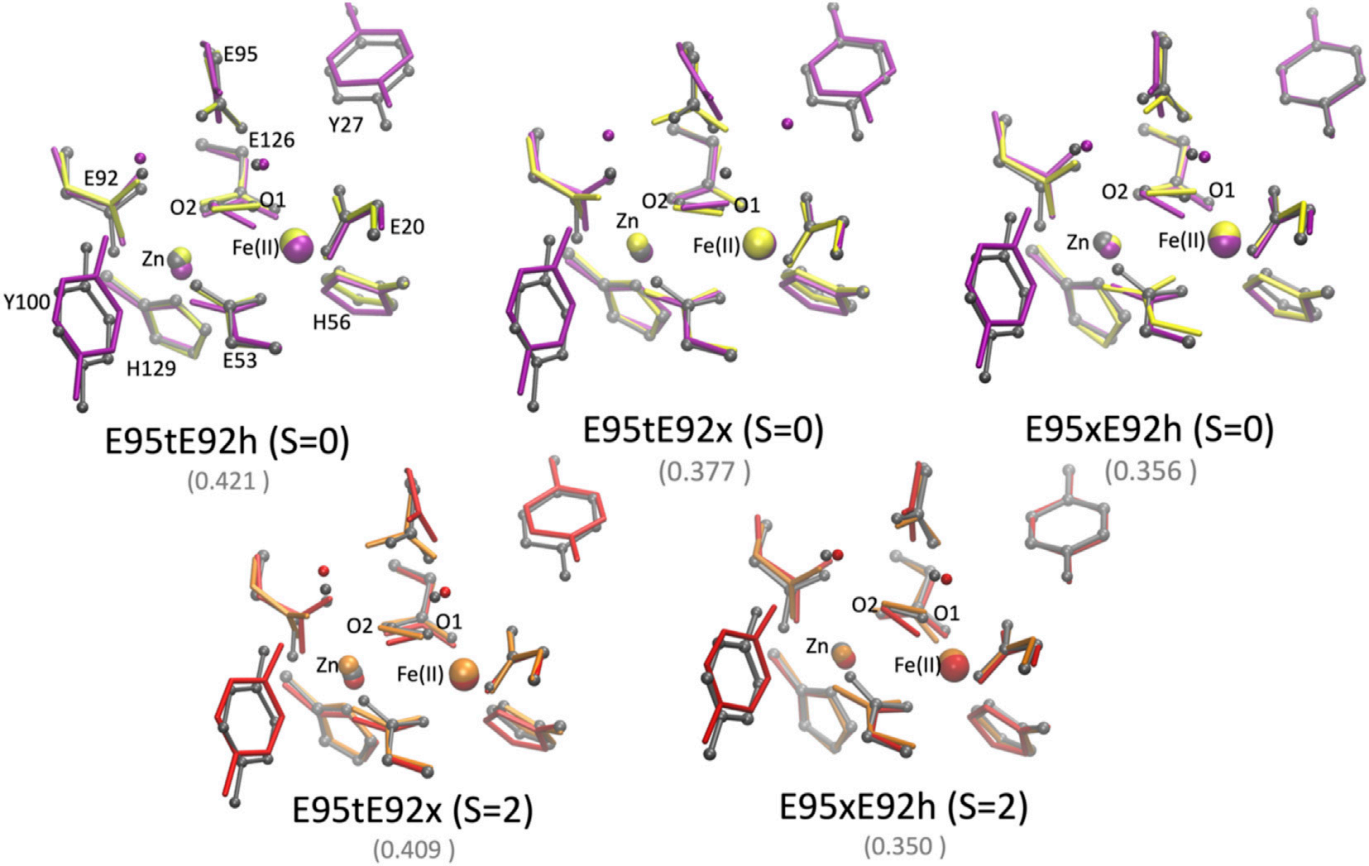
Superposition of the active site of the [ZnFe] SulE crystal structure (gray), B3LYP/def2-TZVP/6-31G* optimized geometries [Fe(II) low spin: yellow; Fe(II) high spin: orange] and QM/MM- optimized geometries [Fe(II) low spin: purple; Fe(II) high spin: red] of the five best structural models E95tE92h_low spin (top, left), E95tE92x_low spin (top, middle), E95xE92h_low spin (top, right), E95tE92x_high spin (bottom, left), and E95xE92h_high spin (bottom, right) with the FeII center harboring an H_2_O_2_ substrate. For clarity, hydrogen atoms are not shown. Corresponding RMSD values listed in Table 8 for the QM/MM models are given in gray fonts.

The QM/MM optimized geometries of all nine models were compared with the [ZnFe] SulE crystal structure and with the corresponding optimized DFT geometries (Figures 8, 9). The RMSD values of the heavy atom positions resulting from optimized QM/MM models, listed in Table 8, differ on the order of 16% from those predicted for the isolated systems described earlier. These more sophisticated computations including electrostatic effects from the protein environment favor the models E95xE92x with low-spin Fe(II) and E95xE92h with high-spin Fe(II) when O_2_ binds to the active site and the E95xE92h with high-spin Fe(II) when H_2_O_2_ is found in the binding pocket, in perfect agreement with the previous DFT predictions. Most of the deviations from the crystal structure predicted for the isolated models also occur in the QM/MM optimized structures such as the significant displacement of the Glu95 in all models with the O_2_ substrate and in the models with protonated Glu95 interacting with the H_2_O_2_ molecule. In addition, minor twists (∼10°) of the bridging bidentate carboxylates from Glu53 and Glu126 as well as slight rearrangement of the dioxygen moiety are predicted in all optimized geometries while the positions of His56 and His129 side chains remain unchanged. The Fe–Zn distances are predicted between 3.78 Å and 4.24 Å very close to the values computed for the isolated systems. In particular, the models harboring H_2_O_2_ with Fe(II) in high spin yield the best agreement with the crystallographic value of 3.83 Å, supporting once again the conclusion derived from the calculations performed on the QM models of the [ZnFe] SulEs catalytic site. Thus, a minimal DFT model including all side chains coordinating the [ZnFe] metal center provides sufficient details for investigating the structural, electronic, and thermodynamic properties of the substrate binding site.

**TABLE 8:** RMSD [Å] of the positions of 39 heavy atoms of the [ZnFeII] active site models (excluding Cα of neighboring side chains and all atoms from the substrate as well as Y27, Y100, w 389, and w302) relative to the crystal structure arrangement [PDB ID 1J30 Fushinobu et al. (2003)] and Zn–Fe distances as predicted for the isolated QM model of the active site and the QM/MM models of SulE.

Model	RMSD		Zn–Fe [Å]	
	QM	QM/MM	QM	QM/MM
E95xE92x_O_2__low spin	0.443	0.366	4.11	4.24
E95tE92x_O_2__high spin	0.450	0.528	4.03	4.09
E95tE92x_O_2__low spin	0.474	0.513	4.10	4.09
E95xE92h_O_2__high spin	0.491	0.405	3.71	3.89
E95tE92h_H_2_O_2__low spin	0.301	0.421	3.79	3.90
E95tE92x_H_2_O_2__high spin	0.480	0.409	3.97	3.84
E95tE92x_H_2_O_2__low spin	0.483	0.377	3.97	3.91
E95xE92h_H_2_O_2__high spin	0.307	0.350	3.79	3.79
E95xE92h_H_2_O_2__low spin	0.361	0.356	3.75	3.78

### Chemical nature of dioxo species

To elucidate the chemical nature of the dioxo ligand detected in the crystal structure of the [ZnFe] SulE reported by Fushinobu et al. (2003), multiple optimized structural models of the catalytic site containing either an O_2_ or a H_2_O_2_ dioxo substrate were analyzed in detail in the previous sections. When the dioxygen moiety is modeled as an O_2_ molecule, according to the QM- and QM/MM calculations, the best geometrical agreement with the crystal structure is predicted for the E95xE92x model in a low FeII spin state and for the E95xE92h model in a high FeII spin state. Among them, the E95xE92x model contradicts the observation that either the glutamate residues or the ligand atom itself is protonated, based on the measured short distances of 2.6 Å between Glu95 and O1/O2 and ∼2.7 Å between Glu92 and O2 of the putative dioxygen-containing species (Fushinobu et al., 2003), respectively, characteristic for H-bond interactions. In addition, a positive interaction energy estimated for this model (∆_I_G = 12 kcal/mol) suggests binding of O_2_ to the [ZnFe] center is unfavorable. In the case of the E95xE92h model in a high FeII spin state, with a protonated Glu92, O_2_ weak binding to the bimetal center is feasible as reflected by interaction energy of −4 kcal/mol. In this model, the O_2_ molecule binds the FeII center *via* an O1 atom in an *end-on* orientation 1.74 Å away from it, while the O2 atom of O2 interacts with the protonated carboxylate side chain Glu92 at a distance of 2.56 Å. All other structural and electronic models converge to a local energy minimum with geometries that significantly differ from the experimental structure.

**FIGURE 10:**
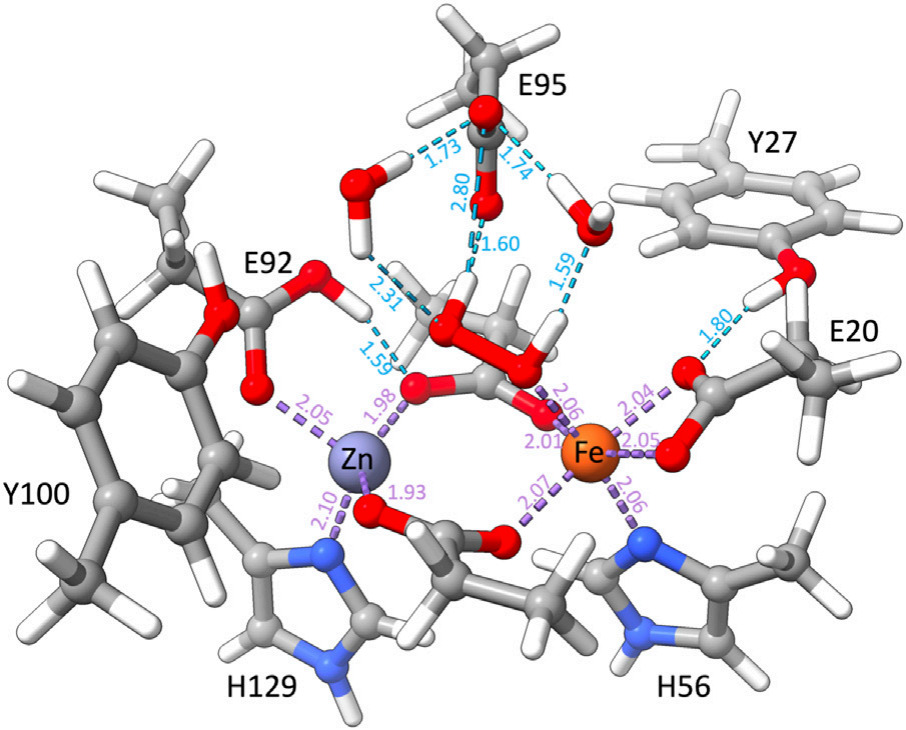
QM/MM optimized structure of the catalytic site of [ZnFe] SulE according to model E95xE92h with Fe(II) in the high-spin state and harboring a H_2_O_2_ molecule. Relevant H-bonds are represented by light blue dashed lines and coordination bonds are depicted as purple dashed lines. Distances are given in Å.

When O_2_ is replaced by H_2_O_2_, QM and QM/MM geometry optimizations of a set of structural and electronic models of the catalytic site with H_2_O_2_ result in structures that barely differ from the crystallographic arrangement. This agreement is particularly true for the FeII E95xE92h models with the so-called “a” conformation of the H_2_O_2_ substrate. This model is characterized by a protonated Glu92 residue and a deprotonated Glu95, both stabilizing the H_2_O_2_ ligand *via* H-bond interactions (Figure 10). Although the structural differences of the two FeII E95xE92h models are negligible, free energy calculations (Table 5) favor the high-spin state of iron(II), computed 33 kcal/mol lower in energy than its low-spin analog, and consistent with reactivity experiments on rubrerythin and studies on electronic properties of non-heme iron complexes (Coulter et al., 1999; Kurtz, 2006).

These results are also in agreement with the experimental data reported by Jeoung et al. (2021) on diCo and diMn SulE variants incubated with H_2_O_2_. For these variants, the measured metal–metal distances of 3.79 Å and 3.74 Å for diMn-SulE and diCo-SulE, respectively (Jeoung et al., 2021), comply with that predicted for the E95xE92h models harboring H_2_O_2_ in “a” conformation (3.79 Å). Nonetheless, our results do not indicate the formation of a bridging (hydro)peroxo ligand as reported for diCo and diMn SulE. In all structural models of [ZnFe] SulE, the H_2_O_2_ binds the Fe center in an *end-on* orientation with one hydrogen atom pointing toward the Glu95 and the other hydrogen atom forming H-bond with water. Hence, these results suggest that [ZnFe] SulE might indeed function similarly to rubrerythrin as a hydrogen peroxidase reductase (Kurtz, 2006; Jeoung et al., 2021).

### Possible reaction pathway at the [ZnFeII] active site of SulE

Since our results suggest an iron(+II) site with a hydrogen peroxide substrate, a possible reaction pathway at the [ZnFeII] active site of SulE characterized by a H_2_O_2_ bound state can be proposed as follows: the electron donor can be either a co-enzyme as it is the case for rubrerythrin co-existing with NAD(P)H or it can be the iron site of the active site of SulE. In the first scenario, the electron donor reacts with SulE and releases a hydrogen atom, resulting in the cleavage of the O–O bond of H_2_O_2_. In the next step, a second electron donor releases another hydrogen atom; a second water molecule is formed. In this manner, hydrogen peroxide can be scavenged *via* a two-electron reduction to water. In a second scenario, the ferrous ion site can act as the electron donor. The ferrous site is oxidized, and hydrogen atoms may be released from bulk solvent or from proximal aromatic residues. Hence, hydrogen peroxide can be scavenged again *via* an electron reduction to water. Independent of the electron donor, a highly oxidizing species, formulated as a hydroxyl radical or a ferryl species ([Fe(IV) = O]^2+^), could arise through the so-called Fenton-type reaction, in which the ferrous iron site is oxidized by H_2_O_2_, forming thus the highly oxidizing species (Kurtz, 2006) Indeed, a ferryl species as an intermediate in the Fenton reaction has been postulated, but its formation has never been documented (Kurtz, 2006). An amino acid with an aromatic side chain, for example, tyrosine, highly conserved in the second coordination sphere, might dissipate the highly oxidative and damaging hydroxyl radical or ferryl species by providing a hydrogen atom, *via* a HAT (hydrogen atom transfer) reaction (Mayer, 2011), thereby reducing the iron site (Kurtz, 2006). The tyrosyl radical may be re-reduced by reactions with the substrate (Kurtz, 2006) or possibly from the bulk solvent (Tommos, 2002) and the ferric ion can be reduced again through the given electron donor. Importantly, both scenarios result in the scavenging of H_2_O_2_, the proposed function of [ZnFe] SulE. Indeed, such peroxidase activity has been recently reported for diCo- and diMn-SulE (Jeoung et al., 2021).

## Conclusion

DFT computational investigations of the active site geometry of [ZnFe] SulE based on available structural data shed light on assumed electronic and structural states of the enzyme. The structural data indicate that the active site contains Zn and Fe metals, though the Zn ions present lower occupancy. Overall, the computed QM optimized geometries for [ZnFe] SulE with an O_2_ substrate show a significant displacement or reorientation of the O_2_ substrate and of the histidine side chains, especially His129, with respect to X-ray data. Although the protein matrix explicitly considered in the QM/MM computations abrogates large conformational distortions, the QM/MM optimized geometries of the active site barely differ from those obtained using the simpler DFT model. The DFT-optimized structural models with H_2_O_2_ as a substrate and an iron(+II) active site are in good agreement with experimentally resolved geometries (Fushinobu et al., 2003; Jeoung et al., 2021), and the presence of two oxygen atoms may be assigned to the H_2_O_2_ substrate. However, DFT models considering an iron(+III) site with a H_2_O_2_ substrate do not describe the structural state well, indicating that this oxidation state may not be favored in [ZnFe] SulE. According to QM/MM calculations, an active site consistent with the E95xE92h model (deprotonated Glu95 and protonated Glu92 side chains) with the H_2_O_2_ substrate in the “a” configuration binding the iron in an *end-on* orientation shows the closest agreement with crystallographic data. Here, a high spin (S = 2) of the Fe(II) is favored over its low-spin (S = 0) analog, as observed in other non-heme iron complexes (Coulter et al., 2000; Jeoung et al., 2021). Interestingly, considering an iron(+II) oxidation level, the “a” configuration of the H_2_O_2_ substrate seems to be more stable than the “b” configuration. Nonetheless, a mixture of the considered models and configurations may be present in a protein sample.

Thus, the proposed reaction pathway for the catalyzed H_2_O_2_ reduction to water through NAD(P)H suggested for rubrerythrin could be extended to [ZnFeII] SulE. However, the C-terminal domain containing a [Fe(Cys)_4_] site, which functions as a motif to transfer reducing equivalents to the diiron site in rubrerythrin (Kurtz, 2006), is lacking in SulE (Wakagi, 2003). A sample in which the iron in the [Fe(Cys)_4_] site from *D. vulgaris* rubrerythrin was artificially and quantitatively substituted with zinc, showed no peroxidase activity (Kurtz, 2006). Hence, this motif seems to be essential for the two-electron reduction of H_2_O_2_ to water through NAD(P)H. Also, the diiron site of rubrerythrin seems to be mandatory for the prevention of Fenton-type chemistry, since the two iron centers within the Fe site of rubrerythrin are nearly identical (Kurtz, 2006). Hence, the Fe site would rather favor two-electron reduction of hydrogen peroxide over mononuclear Fenton-type redox chemistry (Kurtz, 2006). Nonetheless, the residues required for binding the diiron center in the N-terminal domain of anaerobic rubrerythrin are completely conserved in SulE (Wakagi, 2003). Therefore, the [Fe(Cys)_4_] redox center may not be required in an aerobic environment and has been erased for this reason (Wakagi, 2003). Thus, the function of SulE is still not clear (Wakagi, 2003). The calculations in progress investigating other SulE variants will shed light on the possible catalytic mechanisms in SulE and related proteins.

## Data Availability

The raw data supporting the conclusion of this article will be made available by the authors, without undue reservation.
