# Supplementary material for: Structural and electronic properties of the active site of [ZnFe] SulE
Source: Front Mol Biosci. 2022 Oct 10;9:945415. doi: 10.3389/fmolb.2022.945415 (PMC9589262; doi:10.3389/fmolb.2022.945415)
Supplement: Supplementary file 1 [file DataSheet1.PDF]

Supplementary Information

Structural and electronic properties of the active site of [ZnFe] SulE

Samah Moubarak, Yvonne Rippers, Nadia Elghobashi-Meinhardt,
Maria Andrea Mroginski

Table S1: DFT atomic distances for [ZnFe] SulE with O_2 as substrate.
Table S2: DFT O-O bond distances [ZnFe] SulE with O_2 as substrate.
Table S3: DFT atomic distances for [ZnFe] SulE with H_2O_2 as substrate.
Table S4: DFT O-O bond distances [ZnFe] SulE with H_2O_2 as substrate
Table S5: QM/MM atomic distances for [ZnFe] SulE with O_2 as substrate.
Table S6: QM/MM atomic distances for [ZnFe] SulE with H_2O_2 as substrate.

S1. Relevant atomic distances [Å] at the [ZnFe] center of SulE harboring O_2 molecule according to DFT calculations on the isolated model systems. Distances around the experimental values are highlighted.

Model	d_{Zn-Fe} [Å] (exp. 3.83 Å)				d_{Zn-O2} [Å] (exp. 2.74 Å)				d_{Fe-O1} [Å] (exp. 2.12 Å)			
	FeII		FeIII		FeII		FeIII		FeII		FeIII	
	high	low	high	low	high	low	high	low	high	low	high	low
E95xE92x	4.13	4.11	4.16	4.03	3.71	2.99	4.09	3.93	**2.20**	**1.87**	**2.08**	**1.99**
E95tE92x	4.03	4.10	4.37	4.37	3.99	2.93	5.64	3.45	**2.04**	**1.75**	5.15	**2.05**
E95pE92x	4.27	4.12	4.53	4.53	3.90	2.82	4.62	4.64	3.16	**1.75**	3.96	3.99
E95xE92h	**3.71**	**3.73**	**3.63**	-	2.34	**2.31**	3.40	-	2.35	**1.78**	**2.06**	-
E95tE92h	**3.93**	4.06	4.44	4.47	4.69	3.40	4.84	4.87	5.31	**1.74**	4.94	6.00
E95pE92h	**3.96**	**3.89**	4.45	4.47	**2.42**	**2.44**	4.87	4.95	**2.14**	**1.73**	4.95	4.92

S2. O-O bond distances [Å] according to DFT calculations on the isolated model systems.

Model	d_{O-O} [Å] (exp. 1.25 Å)			
	FeII		FeIII	
	high	low	high	low
E95xE92x	1.25	1.27	1.24	1.26
E95tE92x	1.25	1.26	1.21	1.22
E95pE92x	1.21	1.26	1.21	1.21
E95xE92h	1.29	1.29	1.31	-
E95tE92h	1.22	1.29	1.21	1.21
E95pE92h	1.31	1.28	1.21	1.21

S3. Relevant atomic distances [Å] at the [ZnFe] center of SulE harboring H_2O_2 molecule according to DFT calculations on the isolated model systems. Distances around the experimental values are highlighted.

Model	d_{Zn-Fe} [Å] (exp. 3.83 Å)				d_{Zn-O2} [Å] (exp. 2.74 Å)				d_{Fe-O1} [Å] (exp. 2.12 Å)			
	FeII		FeIII		FeII		FeIII		FeII		FeIII	
**Conf. "a"**	high	low	high	low	high	low	high	low	high	low	high	low
E95xE92x	**3.84**	**3.82**	4.26	**3.91**	**2.19**	**2.19**	2.95	**2.31**	**2.05**	**1.98**	**1.93**	**1.84**
E95tE92x	**3.97**	**3.97**	4.14	4.05	**2.56**	**2.59**	**2.40**	**2.40**	2.37	**2.06**	**2.24**	**2.03**
E95xE92h	**3.79**	**3.75**	**3.78**	4.05	3.36	3.32	3.68	3.45	**2.16**	**2.02**	**1.99**	**1.84**
E95tE92h	**3.97**	**3.79**	4.29	4.23	3.53	3.30	3.52	3.56	3.74	**2.13**	2.30	**2.05**
**Conf. "b"**												
E95xE92x	**3.73**	**3.61**	4.14	4.12	**1.97**	**1.99**	2.95	2.79	**2.25**	**2.07**	**2.08**	**1.96**
E95pE92x	4.21	4.13	4.49	4.43	4.33	2.95	3.33	3.15	4.87	**2.07**	2.36	**2.06**
E95xE92h	4.20	**3.97**	**3.89**	4.02	3.73	3.61	3.60	3.74	2.38	**2.04**	**2.19**	4.11
E95pE92h	**3.88**	**3.89**	4.29	4.35	3.73	3.64	3.83	3.32	2.52	**2.10**	2.47	**2.05**

S4. QM O-O bond distances [Å] for high and low spin Fe for all 16 models and both Fe oxidation states at the active site of [ZnFe] sulerythrin with H_2O_2 as substrate.

Model	d_{O-O} [Å] (exp. 1.25 Å)			
	FeII		FeIII	
**Conf. "a"**	high	low	high	low
E95xE92x	1.48	1.48	1.43	1.45
E95tE92x	1.46	1.46	1.45	1.45
E95xE92h	1.47	1.47	1.46	1.46
E95tE92h	1.46	1.47	1.44	1.44
**Conf. "b"**				
E95xE92x	1.47	1.47	1.45	1.46
E95pE92x	1.45	1.46	1.46	1.46
E95xE92h	1.46	1.46	1.44	1.45
E95pE92h	1.45	1.45	1.44	1.45

S5. Relevant atomic distances [Å] of the [ZnFe] center of SulE harboring O_2 molecule according to DFT calculations on the most plausible model systems in vacuum. Distances around the experimental values are highlighted. O-O bond distances are compared to the experimental resolved value of diCo-SulE (exp. 1.50 Å) (PDB 709D). Binding distances around the experimental values are highlighted.

Model	$d_{Zn\text{-}Fe}$ [Å] (exp. 3.83 Å)		$d_{O\text{-}O}$ [Å] (exp. 1.50 Å)		$d_{Zn\text{-}O2}$ [Å] (exp. 2.74 Å)		$d_{Fe\text{-}O1}$ [Å] (exp. 2.12 Å)	
	FeII		FeII		FeII		FeII	
	high	low	high	low	high	low	high	low
E95xE92x	-	4.24	-	1.23	-	3.36	-	**2.19**
E95tE92x	4.09	4.09	1.27	1.27	3.19	3.16	**1.75**	**1.75**
E95xE92h	**3.89**	-	1.27	-	**2.92**	-	**1.74**	-

S6. Relevant atomic distances [Å] at the [ZnFe] center of SulE harboring H_2O_2 molecule according to DFT calculations on the most plausible model systems in vacuum. Distances around the experimental values are highlighted. O-O bond distances are compared to the experimental resolved value of diCo-SulE (exp. 1.50 Å) (PDB 709D). Binding distances around the experimental values are highlighted.

Model	$d_{Zn\text{-}Fe}$ [Å] (exp. 3.83 Å)		$d_{O\text{-}O}$ [Å] (exp. 1.50 Å)		$d_{Zn\text{-}O2}$ [Å] (exp. 2.74 Å)		$d_{Fe\text{-}O1}$ [Å] (exp. 2.12 Å)	
	FeII		FeII		FeII		FeII	
	high	low	high	low	high	low	high	low
E95tE92x_a	**3.84**	**3.91**	**1.45**	**1.45**	3.07	3.43	**2.06**	**2.11**
E95xE92h_a	**3.79**	**3.78**	**1.46**	**1.46**	**2.90**	**2.86**	**2.06**	**2.07**
E95tE92h_a	-	**3.90**	**-**	**1.45**	-	3.14	**-**	**2.10**
